# Re-Vaccination

**Published:** 1902-02-08

**Authors:** Arthur Maude


### Hospital Clinics and Medical Progress.

### RE-VACCINATION.

By Arthur Maude.

(*Continued from page* 303.)

Re-vaccination rarely produces the same objective result as primary vaccination. One of the best clinical descriptions is that of Dr. Gregory, who classifies four different effects of re-vaccination at distant intervals.

1. In many cases, chiefly when the interval from primary vaccination is less than five years, the result is nil.

2. At intervals exceeding ten (10) years the virus irritates locally, producing an areola of irregular shape around a minute, itching, accuminated, and angry vesicle. The glands of the axilla often swell, and in particular habits of body, especially adult female, irritative fever is superinduced. A scab forms which falls off on the eighth day, leaving no permanent cicatrix.

3. Small, well-formed vesicles, with no constitutional disturbance.

4. Perfectly regular course of vaccination.

The points to be accentuated in the above masterly summary are the accuminated form of the pocks, which are rarely plump, full, limpid, and loculated, as in primary vaccination. The contents are rarely clear, they tend to be yellow and turbid. The itching is very pronounced and very annoying, and it usually begins about the second day after vaccination. It is possible that babies may itch as violently after primary vaccination, but are unable to say that they do. The areola in re-vaccination is frequently out of all proportion to the development of the pock. The swelling and painful condition of the axillary glands is proportionately more marked than in primary vaccination. The period of development is very irregular; as a rule it is much accelerated, the vesicles reaching their height by the fifth day, and forming scabs which disappear by the eighth, but the variations in time are very great. On the other hand, the period of development may be greatly prolonged, and this is more often the case after the use of calf lymph. It is unquestionable that re-vaccination does often produce very profound general disturbance in women from the age of 25 to 45. Recently it has been pointed out that the toxic effect of re-vaccination on middle-aged women may be extreme and quite independent of any local septic effect. The writer has seen instances of a disturbance, resembling clinically ptomaine poisoning, occur two or three weeks after re-vaccination of a very mild course in females from 30 to 40 years of age; a subsequent slight anæmia has followed, such as may follow any infective disease however mild.

In the majority of persons the regular phenomena of vaccination can only be produced once in a lifetime; any subsequent introduction of lymph produces one of the modified effects which we have surveyed. It is impossible to say what ratio of good second vaccination we have to expect, as the determining factors are so various—the age of the subject, the time elapsed since the last vaccination, the number of previous vaccinations, the number of successful insertion; but the essentially protective quality of the earlier vaccinations cannot be at all accurately gauged from the number and size of the scars, especially in middle-aged persons, the personal factor in the production of immunity is beyond estimation, and last but not least we have to estimate the effective quality of the lymph employed. Unfortunately as yet no standardisation of lymph has been found possible.

Re-vaccination should immediately be performed on persons who have been in contact with small-pox. Since re-vaccination usually requires but five days to mature, and small-pox has an incubation stage of 12 days, there is ample time for the vaccinia to develop, and the small-pox will be effectively modified, though there is no evidence that vaccination performed after infection can annul the infection. Vaccinia in a small-pox suspect runs a perfectly normal course until the small-pox manifests itself, and even after that may continue undergoing local development, the vaccinia and variola continuing together. But if the vaccination is effected too late, or is retarded, and has not reached its full stage before the small-pox rash appears, it will have no effect in modifying variola.

It is most essential in such a case that the vaccination should not fail; is it possible, however, to ensure against failure? The old vaccinators answered the question. They meant to vaccinate well and their patients meant to be well vaccinated. Both doctors and patients dreaded small-pox and were not afraid of vaccination. They vaccinated in four or preferably five "places" from well-selected arms, but they were not content with this. So good was their intention that they adopted a species of control experiment which should be revived to-day in cases of vaccination after exposure to small-pox infection. Mr. Hicks was a friend of Jenner; he was not a medical man, and his family were the first persons of the upper classes in England who were vaccinated, while Mr. Hicks himself became an expert vaccinator and practised the procedure largely among the poor as a charity. He and Dr. Bryce were in the habit of vaccinating subjects again with a different lymph four or five days after the original vaccination if there was any doubt as to the first insertion being effective. They found that if the first insertion was going to run a proper course the second set of vesicles ran a much more rapid course, "caught up," so to say, the first set, arrived and their height declined and disappeared at the same dates as the first insertion of lymph Further, according to Bousquet, if the first reaction is retarded, or late in development, the second application of lymph seems to revive it, to wake it up, and this has been found to happen even three weeks after the first vaccination.

When an infant has been imperfectly vaccinated, no doubt, the best thing to be done is to vaccinate it again. But if the first vaccination has produced one or two ill-marked vesicles, the second vaccination will probably not produce in any way a proper set of well developed vesicles; the first imperfect vaccination will modify the second. Even if the second vaccination be performed with effective lymph, and it is properly inserted, the combined effect of the two vaccinations is not as satisfactory as one good vaccination. Under the circumstances, undoubtedly the best course would be to re vaccinate again within a few years, but few parents can be persuaded to realise the necessity.

If, however, the result of the primary vaccination be only one or two well-formed vesicles, the failure having been due to careless or faulty insertion, and not to the quality of lymph, Seaton recommends that, except under special conditions of danger, re-vaccination should be deferred till the "child grows up." But most authorities would, we think, now recommend that the next vaccination should be performed somewhat earlier, perhaps at six or seven.

Vaccination is not such a simple performance as it looks, and the proportion of failures in the hands of skilled and practiced vaccinators is far lower than when the practical experience is lacking. If, however, the possibility of failure is present in primary vaccination, it is far more likely to occur in re-vaccination, because such wide modifications exist in results. A contemporary of deserved reputation insists, in a recent leading article, that when a very slight, modified result is obtained in a person not recently vaccinated it is a wise precaution to repeat the inoculation with a different stock of lymph, which has been tested in primary vaccination.

And lastly, what proportion of successful results ought we to anticipate in re-vaccination? The older vaccinators of authority from Jenner to Seaton will not rashly commit themselves to figures: It is impossible to do so. The factors which are concerned are so many, the age of the person, the quality of the first vaccination, the value of the lymph employed in re-vaccination, and the quite unaccountable factor of the degree of immunity of each individual; all these render an answer impossible. The statistics, numerous enough, afforded by re-vaccination of troops do not apply to a mixed population of all ages, for the soldiers, whether recruits or long service men, are much more of an age and the quality of lymph employed on them more of one value, than are the ages of a civil population and the lymph applied to them.

